# Disordered eating behaviour in adolescents with type 1 diabetes on continuous subcutaneous insulin infusion; relation to body image, depression and glycemic control

**DOI:** 10.1186/s40337-022-00571-4

**Published:** 2022-04-04

**Authors:** Nouran Yousef Salah, Mostafa Ahmad Hashim, Mai Seif ElDin Abdeen

**Affiliations:** 1 Department of Pediatrics, Faculty of Medicine, Ain Shams University, 25 Korash Street, Nasr City, Cairo, Egypt; 2 Department of Psychiatry, Faculty of Medicine, Ain Shams University, Cairo, Egypt

**Keywords:** Adolescents, Type 1-diabetes, Continuous subcutaneous insulin infusion, Body image, Eating disorders

## Abstract

**Background:**

Disordered eating behaviour (DEB) represents a significant morbidity among people with type-1 diabetes (T1D). Continuous-subcutaneous insulin infusion (CSII) improves glycemic control and psychological wellbeing in those with T1D. However, its relation to DEB remains obscure.

**Objectives:**

To compare DEB among adolescents with T1D on CSII versus basal-bolus regimen and correlate it with body image, HbA1C and depression.

**Methods:**

Sixty adolescents with T1D (30 on CSII and 30 on basal-bolus regimen), aged 12–17 years were studied focusing on diabetes-duration, insulin therapy, exercise, socioeconomic standard, hypoglycemic attacks/week and family history of psychiatric illness. Anthropometric measures, HbA1C, binge eating scale (BES), body image tool, patient health questionnaire-9 (PHQ9) and the Mini-KID depression scale were assessed.

**Results:**

Among the studied adolescents with T1D, six had DEB (10%), 14 had poor body-image perception (23.3%), 42 had moderate body-image perception (70%) and 22 had depression (36.7%). Adolescents with T1D on CSII had significantly lower BES (*p* = 0.022), Mini-KID depression (*p* = 0.001) and PHQ9 (*p* = 0.02) than those on basal-bolus regimen. BES was positively correlated to depression (*p* < 0.001), HbA1C (*p* = 0.013) and diabetes-duration (*p* = 0.009) and negatively correlated to body-image (*p* = 0.003).

**Conclusion:**

DEB is a prevalent comorbidity among adolescents with T1D, with higher frequency in those on basal-bolus regimen than CSII.

## Plain English summary

Disordered eating behaviour is a significant morbidity among people with type 1 diabetes. It is associated with poor metabolic control and diabetes related complications even when the full diagnostic criteria of an eating disorder are not met. There is conflicting data in the literature regarding the prevalence of disordered eating behaviour in type 1 diabetes. The current study found that disordered eating behaviour is prevalent among 10% of the studied adolescents with type 1 diabetes. It was found to be more severe and frequent among those on basal-bolus insulin regimen than those on continuous subcutaneous insulin infusion. Moreover, it was correlated with depression, poor glycemic control, poor body image and long diabetes duration. Thus further studies are needed to verify the role of continuous glucose monitoring in the management of DEB among those with T1D.

## Highlights

- Disordered eating behavior is a prevalent morbidity among adolescents with type 1 diabetes.
- Binge eating scale is positively correlated with depression, HbA1C and diabetes duration and negatively correlated with body image.
- Adolescents with type 1 diabetes on continuous subcutaneous insulin infusion have significantly lower frequency and severity of disordered eating behaviors.

## Introduction

Disordered eating behaviour (DEB) represents a significant comorbidity among people with type 1 diabetes (T1D) [1, 2]. It is associated with poor metabolic control and diabetic complications even when the full diagnostic criteria of an eating disorder are not met [3]. DEB typically begins during adolescence and early adulthood [4]. As in the general population, DEB in adolescents with T1D is associated with higher BMI *z* score, younger age, female gender and body image distortion. In addition, specific behaviours and attitudes related to diabetes, such as dietary restriction, counting carbohydrates and insulin injection impose an additional risk for DEB [5].

Multiple risk factors are associated with DEB in adolescents with T1D. This includes emotional factors and disrupted hunger and satiety balance. People with diabetes have higher levels of depression and anxiety than do people in the general population [6]. Depression is a known risk factor for DEB and body image distortion [7]. Hypoglycemia, a common symptom of uncontrolled diabetes disrupts self-feeding regulation, leading to increased hunger and loss of control over eating among adolescents with T1D [8].

Treatment modalities of T1D may also influence DEB. The use of continuous subcutaneous insulin infusion (CSII) is rising among children and adolescents with T1D. Studies document the beneficial role of CSII on glycemic control, quality of life and psychological wellbeing [9]. However, data about the impact of CSII on DEB are scarce and contradictory. The use of CSII may either improve DEB or worsen it [10]. On one hand, CSII imposes more focus on food intake and carbohydrate counting. On the other hand, it gives better flexibility in meal timing and contents decreasing diabetes distress and negative affect related to feeding. People on basal-bolus regimen have more depressive symptoms than those on CSII. However the relation between DEB and affect in people on CSII compared to basal-bolus is unknown.

Conventional eating disorder therapies are less effective for people with T1D [7]. DEB persists in adolescents with T1D even when there is improvement in body image [11]. This highlights the importance of identifying the unique risk determinants for DEB among adolescents with T1D in order to develop effective interventional modalities for them.

## Aim

The aim of this study is to compare DEB in adolescents with T1D on CSII versus basal-bolus regimen and to correlate it with body image, glycemic control, and depressive symptoms.

## Methodology

### Study design and setting

This cross sectional study included sixty adolescents with T1D recruited from the Pediatric Diabetes Clinic, Pediatric Hospital, Ain Shams University during the period from December 2020 to March 2021. The study protocol was approved by the Ethical Committee of Ain Shams University, and an informed consent was obtained from each patient or their legal guardians before participation. Reporting of the study conforms to Consolidated Standards of Reporting Trials 2010 statement [12].

### Patients' selection

Inclusion criteria included adolescents with T1D according to the International Society of Pediatric and Adolescents Diabetes (ISPAD) 2018 [13], aged 10–18 years on daily insulin therapy with CSII or basal-bolus regimen for at least 1 year. They were classified into two groups according to the mode of insulin administration. The selection for insulin therapy type (CSII or basal-bolus) was based on parental/patient factors including affordability/social class and patient/parent preference. Adolescents with comorbid conditions (i.e. celiac disease or autoimmune thyroiditis), other types of diabetes (i.e. maturity onset diabetes of youth [MODY] and type 2 diabetes mellitus) and those with history of psychiatric disorders were excluded.

### Clinical assessment

All included adolescents were subjected to detailed medical history with special emphasis on disease duration, insulin daily dose (U/kg/day), exercise and the presence of clinically significant hypoglycemic episodes “i.e. glucose value of < 3.0 mmol/l (54 mg/dl)/week” [14]. The socioeconomic status was assessed using the validated Arabic socioeconomic status scale for health research in Egypt. It is a scale with 7 domains with a total score of 84. According to the scale the socioeconomic level is classified into very low, low, middle and high levels depending on the quartiles of the score calculated [15].

Thorough clinical examination was done laying stress on anthropometric measures and body mass index (BMI) measured as kg/m2 with calculation of z score [16]. Peripheral blood samples were collected on potassium-ethylene diamine tetra-acetic acid (K2-EDTA) in sterile vacutainer tubes (final concentration of 1.5 mg/ml) (Beckton Dickinson, Franklin Lakes, NJ, USA) for assessment of HbA1C.

### Psychological assessment

Family history of psychiatric illness was assessed by self report due to the lack of registration files. The severity of depressive symptoms was assessed with the Arabic version [17] of the nine item patient health questionnaire (PHQ-9) [18]. This self report questionnaire includes the nine symptoms of the DSM-IV criteria for a major depressive disorder. A score of 5–9 is considered mild, 10–14 is moderate, 15–19 is moderately severe and ≥ 20 is severe depression. PHQ-9 demonstrates acceptable reliability and is validated among diabetes patients [19]. Though initially developed as a depression screening tool for adults, recent studies have shown that it is a reliable and valid tool for detection of depression among adolescents [20, 21].

A validated Arabic version [22] of the Mini International Neuropsychiatric Interview for Children and Adolescents (MINI-KID), depression module was used as diagnostic test for depression. The MINI-KID provides a structured interview for DSM IV and ICD-10 childhood and adolescent disorders [23].

For assessment of DEB, the binge eating scale (BES) Arabic version was used [24]. It is a 16-item self-reporting questionnaire designed to capture the behavioural, as well as the cognitive and emotional features of DEB in adults [25]. For each item, respondents are asked to select one of three or four response options, coded zero to two or three, respectively. Individuals’ scores are summed and ranged from 0 to 46, with higher scores indicating more severe binge eating problems. Clinical cut-off scores for the BES represent none-to-minimal (≤ 17 total score), moderate (18–26), and severe (> 27) binge eating problems. DEB was defined as having a BES score of > 17 [25]. The BES has been validated for use in children and adolescents [26].

The body image tool (an Arabic questionnaire that consists of a 34-item scale with scores between 1 [never] to 5 [always] for each item) was used for detection of body image distortion [27]. It measures the cognitive and behavioural symptoms associated with personal body dissatisfaction. A lower score less than 112 indicates more body dissatisfaction while higher score more than 140 indicates high self-body image satisfaction and between 112 and 140 scores indicates average body satisfaction [28].

### Statistical analysis

Analysis of data was performed using software MedCalc v. 19. Data were explored for normality using Kolmogorov–Smirnov test of normality. Quantitative data were presented as mean, standard deviations and ranges when their distribution was normally distributed and median with inter-quartile range (IQR) when their distribution was non parametric. Qualitative variables were presented as frequency and percentage. The comparison between groups with qualitative data was done by using Chi-square test when more than 20% of cells have expected frequencies ≥ 5 and with Fisher exact test when more than 20% of cells have expected frequencies < 5. Comparison between data with parametric distribution was done using independent t-test while for non-parametric data this was done by using the Mann–Whitney test. Binary correlation was carried out by Pearson correlation test. Results were expressed in the form of correlation coefficient (R) and *p* values. The correlation coefficient was interpreted as no linear relationship (0), a perfect positive linear relationship (+ 1) and a perfect negative linear relationship (− 1). Values between 0 and 0.3 or (0 and − 0.3) indicate no or a weak positive (negative) linear relationship. Values between 0.3 and 0.7 or (− 0.3 and − 0.7) indicate a moderate positive (negative) linear relationship. Values between 0.7 and 1.0 or (− 0.7 and − 1.0) indicate a strong positive (negative) linear relationship. Multivariate linear regression analysis was used to assess predictors of DEB among the studied adolescents with T1D. The confidence interval was set to 95% and the margin of error accepted was set to 5%, so the *p* value was considered significant at a level of < 0.05.

## Results

The mean age of the studied adolescents with T1D was 13.35 ± 3.28 years. They were 35 females (58.3%) and 25 males (41.7%). Their median BMI was − 0.08 (− 1.05 to 0.74) and their mean HbA1C was 8.49%, range 6.6–13.5.

### DEB in adolescents with T1D

Six adolescents with T1D were found to have DEB (10%). The median BES of the studied adolescents with T1D was 7, range 0–22. Their median body image tool was 121.5, range 84–145, with fourteen having poor body image perception (23.3%), forty two having moderate body image perception (70%) and only four having good body image perception (6.7%). Twenty two adolescents with T1D had depression (36.7%).

### DEB and CSII

None of the adolescents with DEB or poor body image perception was on the CSII regimen. Adolescents with T1D on CSII had significantly lower BES (*p* = 0.022), Mini-kid depression scale (*p* = 0.001) and PHQ9 (*p* = 0.02) with significantly higher socioeconomic status scale (*p* = 0.041) than those on basal-bolus regimen, Table 1.

**Table 1:** Comparison of the clinico-demographic data between the studied children with T1D on basal bolus regimen and CSII

	Basal bolus group N = 30	CSII group N = 30	Test value	*p* value
Age (years)				
Mean ± SD	12.10 ± 1.75	12.96 ± 1.35	0.330•	0.742
Range	12–17	12–16		
Gender				
Females	17 (56.7%)	18 (60.0%)	0.069*	0.793
Males	13 (43.3%)	12 (40.0%)		
Smoking				
Negative	28 (93.3%)	30 (100.0%)	2.069*	0.150
Positive	2 (6.7%)	0 (0.0%)		
Exercise/week				
Negative	29 (96.7%)	21 (70.0%)	7.680*	**0.006**
Positive	1 (3.3%)	9 (30.0%)		
Family history of psychiatric disease				
Negative	28 (93.3%)	30 (100.0%)	2.069*	0.150
Positive	2 (6.7%)	0 (0.0%)		
Diabetes duration (years)				
Mean ± SD	4.53 ± 1.87	4.29 ± 1.73	0.513•	0.610
Range	1–7	1–7		
Socioeconomic status scale				
Mean ± SD	56.80 ± 2.89	58.90 ± 4.69	− 2.089•	**0.041**
Range	44–60	50–68		
Height z-score				
Median (IQR)	− 0.55 (− 0.96 to 0.88)	− 0.86 (− 1.13 to 0.93)	− 0.043 ≠	0.966
Range	− 1.5 to 1.17	− 1.2 to 1.81		
Weight z-score				
Median (IQR)	− 0.07 (− 0.91 to 0.55)	− 0.37 (− 0.86 to 0.87)	− 0.085 ≠	0.932
Range	− 1.03 to 1.34	− 2 to 1.71		
BMI z-score				
Median (IQR)	0.11 (− 0.56 to 0.75)	− 0.08 (− 1.07 to 0.38)	− 0.468 ≠	0.639
Range	− 1.02 to 1.35	− 2 to 2		
Total daily insulin dose (U/kg/day)				
Mean ± SD	1.09 ± 0.33	0.86 ± 0.29	0.204•	**0.007**
Range	0.8–2.0	0.8–1.8		
HbA1C (%)				
Mean ± SD	9.10 ± 1.94	7.88 ± 1.35	2.809•	**0.007**
Range	6.2–13.5	5.6–10.3		
Frequency of hypoglycemic attacks/week				
Negative	25 (83.3%)	25 (83.3%)	1.111*	0.574
Positive	5 (16.6%)	5 (16.7%)		
PHQ9				
Median (IQR)	7 (6–9)	5 (3–7)	2.327 ≠	**0.020**
Range	2–27	1–16		
Mini-Kid				
Depression	17 (56.7%)	5 (16.7%)	10.163*	**0.001**
No depression	13 (43.3%)	25 (83.3%)		
BES				
Median (IQR)	10.0 (4–15)	5 (4–8)	− 2.283 ≠	**0.022**
Range	0–22	1–19		
Body image tool				
Median (IQR)	125 (106–129)	120 (115–130)	0.440 ≠	0.660
Range	84–138	88–145		

Bold: significant

*T1D* type 1 diabetes mellitus, *CSII* continuous subcutaneous insulin infusion, *BMI* body mass index, *PHQ* patient health questionnaire, *Mini-Kid* Mini International Neuropsychiatric Interview for Children and Adolescents, *BES* binge eating scale

*p* value > 0.05: non significant; *p* value < 0.05: significant; *p* value < 0.01: highly significant

*: Chi-square test; •: Independent t-test; ≠ : Mann–Whitney test

### DEB risk determinants

Upon comparing adolescent with T1D having DEB and those without, DEB was significantly lower in those on CSII (*p* = 0.009) than those on basal-bolus regimen. Moreover, it was positively related to family history of psychiatric disease (*p* = 0.008), smoking (*p* = 0.008), HbA1C (*p* = 0.038) and diabetes duration (*p* = 0.028), depression (*p* = 0.004) and poor body image (*p* = 0.003), Table 2. Although DEB was more prevalent in females, no significant relation was found between DEB and gender (*p* = 0.190). Notably, no significant relation was found between the socioeconomic status and DEB (*p* = 0.634) despite being significantly lower among those on CSII.

**Table 2:** Comparison between adolescents with T1D with and without DEB

	DEB		Test value	*p* value
	Negative N = 54	Positive N = 6		
Age (years)				
Mean ± SD	12.00 ± 1.57	12.33 ± 1.51	0.496^•^	0.622
Range	12–17	12–15		
Gender				
Female	30 (55.6%)	5 (83.3%)	1.714*	0.190
Male	24 (44.4%)	1 (16.7%)		
Smoking				
Negative	54 (100.0%)	4 (66.7%)	F°	**0.008**
Positive	0 (0.0%)	2 (33.3%)		
Exercise/week				
Negative	46 (85.2%)	4 (66.7%)	1.333*	0.248
Positive	8 (14.8%)	2 (33.3%)		
Family history of psychiatric disease				
Negative	54 (100.0%)	4 (66.7%)	F°	**0.008**
Positive	0 (0.0%)	2 (33.3%)		
Disease duration (years)				
Mean ± SD	3.61 ± 1.31	4.87 ± 1.25	2.244^•^	**0.028**
Range	1–7	2–7		
Socioeconomic status scale				
Mean ± SD	58.18 ± 4.53	57.27 ± 2.90	0.479^•^	0.634
Range	44–68	50–63		
Wt Z score				
Median (IQR)	− 0.09 (− 0.92 to 0.52)	− 0.69 (− 0.95 to 0.11)	− 0.789^≠^	0.430
Range	− 2 to 1.71	− 1.03 to 1.14		
Height Z score				
Median (IQR)	− 0.83 (− 1.16 to − 0.01)	− 1.18 (− 1.43 to − 0.44)	− 1.417^≠^	0.156
Range	− 1.5 to 1.81	− 1.5 to − 0.08		
BMI Z score				
Median (IQR)	0.56 (0.08–1.15)	0.38 (− 0.08 to 0.56)	− 0.345^≠^	0.730
Range	− 2 to 2	− 0.24 to 1.96		
HbA1C %				
Mean ± SD	7.47 ± 1.51	8.82 ± 1.09	2.122^•^	**0.038**
Range	5.6–13.5	7.2–10.5		
Mode of treatment				
Basal bolus	24 (44.4%)	6 (100.0%)	6.667*	**0.009**
CSII	30 (55.6%)	0 (0.0%)		
Total insulin daily dose (U/kg day)				
Mean ± SD	1.05 ± 0.28	1.03 ± 0.15	0.188^•^	0.851
Range	0.8–2	0.9–1.3		
PHQ9				
Median (IQR)	6 (3–8)	11 (10–12)	− 2.851^≠^	**0.004**
Range	1–16	6–27		
Body image tool				
Median (IQR)	126.5 (116–132)	97 (89–99)	− 2.942^≠^	**0.003**
Range	88–145	84–119		

Bold: significant

*T1D* type 1 diabetes mellitus, *CSII* continuous subcutaneous insulin infusion, *BMI* body mass index, *PHQ* patient health questionnaire, *Mini-Kid* Mini International Neuropsychiatric Interview for Children and Adolescents, *DEB* disordered eating behaviour

*p* value > 0.05: non significant; *p* value < 0.05: significant; *p* value < 0.01: highly significant

*: Chi-square test; ° Fisher exact test; •: independent t-test; ≠ : Mann–Whitney test

**Fig. 1:**
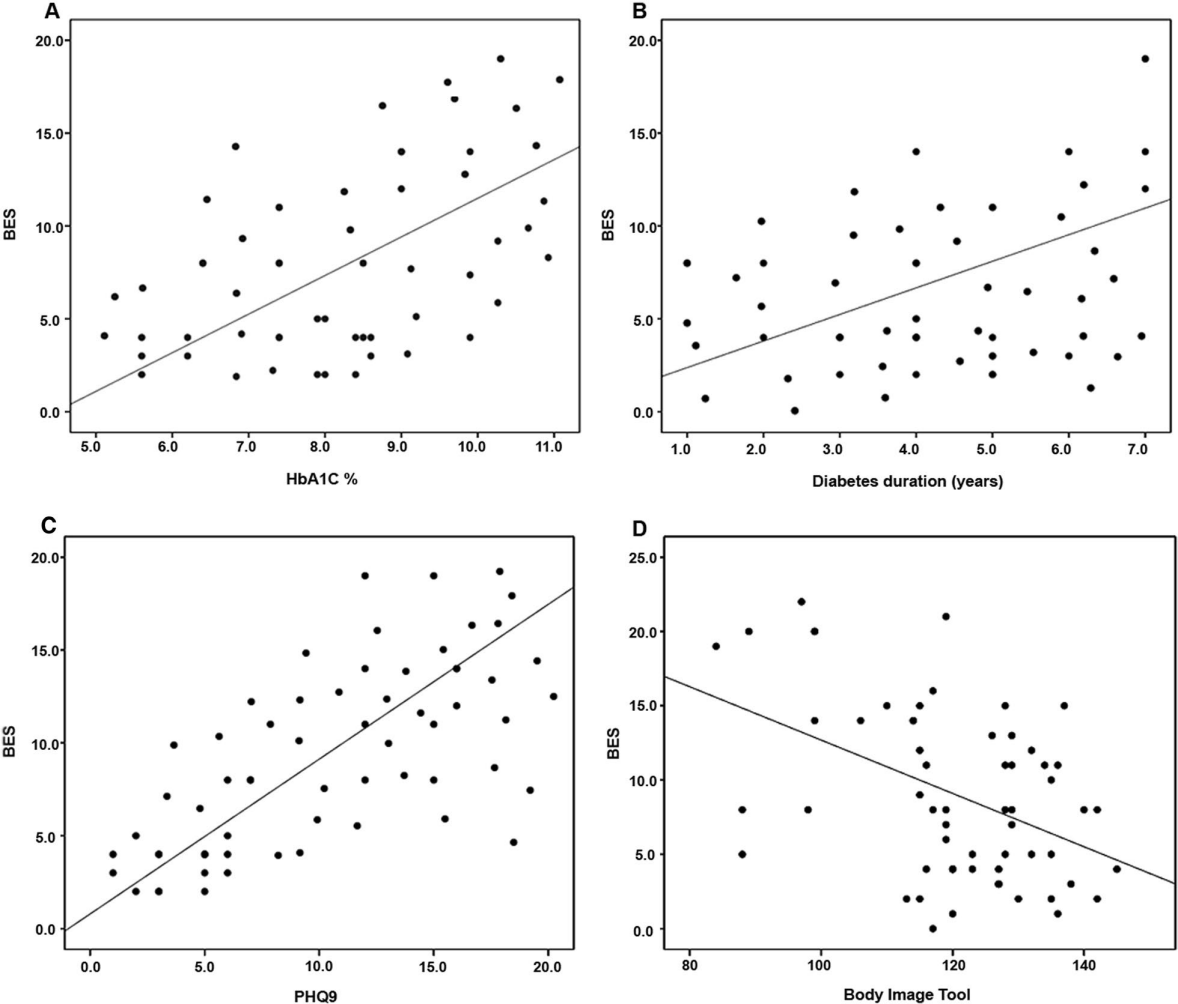
Scatter plot showing correlation between BES scores and each of **A** HbA1C value, **B** diabetes duration, **C** PHQ9 score and **D** body image tool score

Correlations revealed that BES was positively correlated to PHQ9 (*p* < 0.001), HbA1C (*p* = 0.013) and diabetes duration (*p* = 0.009) and negatively correlated to body image (*p* = 0.003), Fig. 1.

Multi-variate logistic regression analysis for predictors of DEB revealed that it was most correlated to the disease duration (*p* = 0.037) and body image (*p* = 0.02), Table 3.

**Table 3:** Multivariate linear regression analysis for factors independently associated with DEB among the studied adolescents with T1D

	Unstandardized coefficients		Standardized coefficients	t	Sig
	B	Std. error	Beta		
(Constant)	9.355	7.977		1.173	0.246
Disease duration (years)	0.893	0.418	0.240	**2.136**	**0.037**
HbA1C%	0.762	0.406	0.208	1.875	0.066
PHQ9	0.292	0.153	0.225	1.916	0.061
Body image tool	− 0.114	0.047	− 0.284	**− 2.399**	**0.020**

Bold: significant

*T1D* type 1 diabetes mellitus, *PHQ* patient health questionnaire, *DEB* disordered eating behaviour

*p* value > 0.05: non significant; *p* value < 0.05: significant; *p* value < 0.01: highly significant

## Discussion

Despite the prevalence and clinical significance of DEB in T1D, it remains understudied and effective treatment modalities are lacking. Adolescents with T1D are especially vulnerable to DEB, since both adolescence and diabetes impose risk factors for DEB [29]. Moreover, the co-occurrence of DEB and T1D dramatically increase the rates of morbidity and mortality [30]. Hence, understanding the risk determinants of DEB among adolescents with T1D is crucial to identify effective treatment modalities.

The prevalence of DEB in the studied cohort of adolescents with T1D was 10%. This prevalence is lower than previous Danish and Italian studies that found DEB in 21% and 21.8% of adolescents with T1D, respectively [31, 32]. Prevalence estimates for DEB are variable between different studies with a wide range from < 1 to 39% [33–36]. This discrepancy could be attributed to cultural differences, different study designs, timing and sample characteristics among the studied populations.

The association between T1D and DEB could be attributed to the presence of several risk factors for DEB in T1D, like lifelong insulin therapy, attendant weight gain, food preoccupation (e.g., carbohydrate counting), low self‐esteem, and depression [37]. Weight gain during puberty is exacerbated in adolescents with T1D on intensified insulin therapy which can lead to DEB [38].

Peterson and colleagues, 2018 hypothesized a modified dual pathway model to determine the risk factors of developing DEB in people with T1D. They hypothesized that diabetes duration, disruption to hunger and satiety secondary to exogenous insulin administration and fluctuations in blood glucose increase the risk for DEB in people with T1D [8]. The preceding goes in line with the current results where adolescents with T1D having DEB had significantly higher diabetes duration and HbA1C than those without DEB. Similarly, Nip and coworkers found that HbA1C was higher in youth with T1D with DEB than those without [5].

The effect of glycemic and insulin fluctuations on hunger and satiety was recently proven by Al-Zubaidi et al. who found that glycemic and insulin fluctuations in healthy normal weighed men were associated with changes in the brain hunger and satiety centers activity [39]. The poor metabolic control associated with DEB in people with T1D could explain their higher vulnerability to diabetic complications.

DEB among adolescents with T1D was significantly associated with higher depression scale, poor body image and family history of psychological disorders. Depression is a well-known risk factor of DEB in people with T1D as well as in the general population [40].

Interestingly, no significant relation was found between DEB among the studied adolescents with T1D and gender. However, this need to be further verified as they might be underpowered with only 25 males in the study. Research on DEB in T1D often targets female adolescents and young adults. So far, men have been underrepresented in the DEB literature. Recent research suggests that DEB is not as rare in men as it has been assumed [41]. In agreement with the current results, a study using a diabetes-specific DEB questionnaire found a similar rate of DEB in males and females with T1D [42].

Treatment modality for T1D might influence DEB in people with T1D. In the current study adolescents with T1D on CSII had significantly lower prevalence of DEB than those on basal-bolus regimen. Previous data about the relation between DEB and CSII are contradictory and mostly come from adult studies. A study by Markowitz et al. suggests that DEB improved following treatment with CSII [10]. Similarly, a retrospective pilot study by Pinhas-Hamiel and colleagues showed that DEB was less prevalent among adolescent females on CSII compared with those on basal-bolus regimen [43]. In agreement with these results, Sanlier and colleagues reported lower depression and eating disorders survey scores for those using CSII (n = 24) in a sample of 149 children and adolescents with T1D [44]. In contrast, Prinz et al. found no significant difference in the frequency of DEB between those on CSII and basal-bolus regimen. However, they found that the rate of CSII discontinuation tends to be higher among people with T1DM and co-morbid DEB [45]. The lower frequency of DEB in those on CSII might be attributed to the improved eating behaviour in those on CSII given the higher flexibility in one's diabetes management, the reduced daily insulin requirements leading to a less pronounced weight gain, the better affect and glycemic control compared with basal-bolus regimen.

One limitation of this study is its relatively small sample size; its cross-sectional nature and the lack of randomization to different treatments which undermines its ability to draw causal relations. Therefore, further studies are needed to identify the effect of CSII on DEB among adolescents with T1D.

In conclusion, DEB is a prevalent morbidity among adolescents with T1D. It is associated with depression, distorted body image and poor glycemic control. Adolescents with T1D on CSII have significantly lower frequency and severity of DEB. Thus, CSII could be a promising treatment modality for adolescents with T1D having DEB. Further larger longitudinal studies are needed to verify the practical utility of CSII and the role of continuous glucose monitoring in the management of DEB among those with T1D.

## Data Availability

Data will be available upon request.
